# Targeting Multiresistant Gram-Positive Bacteria by Ruthenium, Osmium, Iridium and Rhodium Half-Sandwich Type Complexes With Bidentate Monosaccharide Ligands

**DOI:** 10.3389/fchem.2022.868234

**Published:** 2022-04-12

**Authors:** Bence Balázs, Zoltán Tóth, István Kacsir, Adrienn Sipos, Péter Buglyó, László Somsák, Éva Bokor, Gábor Kardos, Péter Bai

**Affiliations:** ^1^ Department of Metagenomics, University of Debrecen, Debrecen, Hungary; ^2^ Department of Organic Chemistry, University of Debrecen, Debrecen, Hungary; ^3^ Doctoral School of Chemistry, University of Debrecen, Debrecen, Hungary; ^4^ Department of Medical Chemistry, Faculty of Medicine, University of Debrecen, Debrecen, Hungary; ^5^ Department of Inorganic and Analytical Chemistry, Faculty of Sciences and Technology, University of Debrecen, Debrecen, Hungary; ^6^ NKFIH-DE Lendület Laboratory of Cellular Metabolism, Debrecen, Hungary; ^7^ Research Center for Molecular Medicine, Faculty of Medicine, University of Debrecen, Debrecen, Hungary

**Keywords:** platinum-group metal complexes, half-sandwich, glycosyl heterocycle, oxadiazole, triazole, gram positive, MRSA, VRE

## Abstract

Bacterial resistance to antibiotics is an ever-growing problem in heathcare. We have previously identified a set of osmium(II), ruthenium(II), iridium(III) and rhodium(III) half-sandwich type complexes with bidentate monosaccharide ligands possessing cytostatic properties against carcinoma, lymphoma and sarcoma cells with low micromolar or submicromolar IC_50_ values. Importantly, these complexes were not active on primary, non-transformed cells. These complexes have now been assessed as to their antimicrobial properties and found to be potent inhibitors of the growth of reference strains of *Staphylococcus aureus* and *Enterococcus faecalis* (Gram-positive species), though the compounds proved inactive on reference strains of *Pseudomonas aerugonisa, Escherichia coli, Candida albicans, Candida auris* and *Acinetobacter baumannii* (Gram-negative species and fungi). Furthermore, clinical isolates of *Staphylococcus aureus* and *Enterococcus* sp. (both multiresistant and susceptible strains) were also susceptible to the organometallic complexes in this study with similar MIC values as the reference strains. Taken together, we identified a set of osmium(II), ruthenium(II), iridium(III) and rhodium(III) half-sandwich type antineoplastic organometallic complexes which also have antimicrobial activity among Gram-positive bacteria. These compounds represent a novel class of antimicrobial agents that are not detoxified by multiresistant bacteria suggesting a potential to be used to combat multiresistant infections.

## Introduction

Bacterial resistance to registered antibiotics is one of the biggest challenges of mankind (Hernando-Amado et al., 2019; Murray et al., 2022) that begs for the discovery of novel antibacterial compounds. There are multiple examples of antibacterial agents that were repurposed as anticancer drugs [e.g., Methenamine (Altinoz et al., 2019)], or anticancer medications being repurposed as antibacterial ones, such as platinum(II) remedies. Indeed, cisplatin and carboplatin do have bacteriostatic properties on *Acinetobacter*, *Mycobacteria*, and *Pseudomonas aeruginosa* (Zhang et al., 2011; McCarron et al., 2012; Yuan et al., 2018) and other pathogens (Hummell and Kirienko, 2020). To complement the registered platinum-based anticancer agents, there is a thrust towards identifying novel complexes of transition metals with anticancer activity (Kenny and Marmion, 2019). Ruthenium complexes have emerged as anticancer agents, characterized by low toxicity (Melchart and Sadler, 2006; Mello-Andrade et al., 2018; Gano et al., 2019; Liu et al., 2019; Mihajlovic et al., 2020), good cellular entry properties (Graf and Lippard, 2012; Yadav et al., 2013) and with excellent targetability (Berger et al., 2008; Hanif et al., 2013; Florindo et al., 2014; Zeng et al., 2017; Kenny and Marmion, 2019; Hamala et al., 2020). In fact, a ruthenium complex, IT-139 has passed clinical phase I to be applied in colorectal cancer (Burris et al., 2016). Furthermore, rhodium (Leung et al., 2013; Gichumbi and Friedrich, 2018; Štarha and Trávníček, 2019; Máliková et al., 2021), osmium (Hartinger et al., 2011; Hanif et al., 2014; Gichumbi and Friedrich, 2018; Konkankit et al., 2018; Meier-Menches et al., 2018; Štarha and Trávníček, 2019; Nabiyeva et al., 2020; Li et al., 2021) and iridium (Leung et al., 2013; Liu and Sadler, 2014; Gichumbi and Friedrich, 2018; Konkankit et al., 2018; Štarha and Trávníček, 2019; Li et al., 2021) compounds were also described as anticancer agent candidates.

**FIGURE 1:**
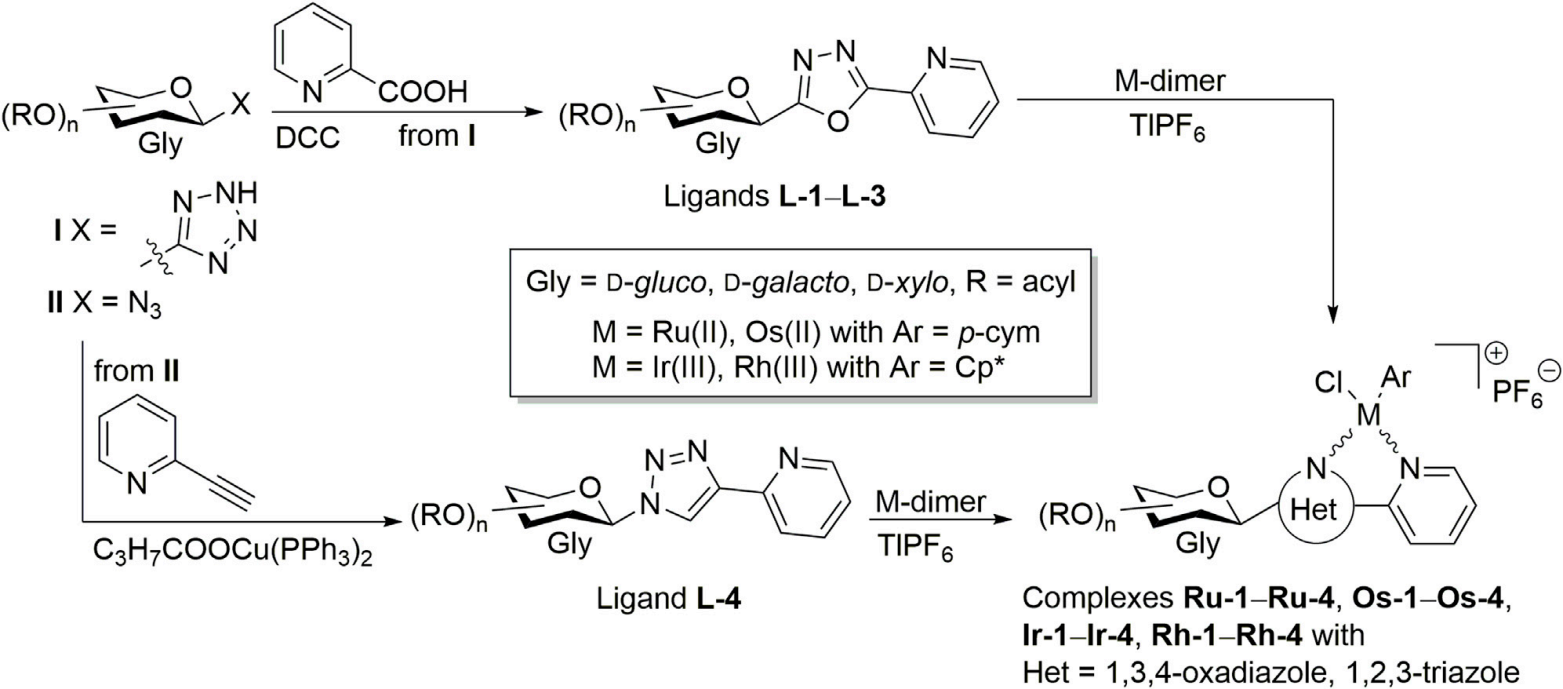
Outline of the syntheses of the compounds to be tested in this study (the precise structures of the compounds are shown in Figure 2).

**FIGURE 2:**
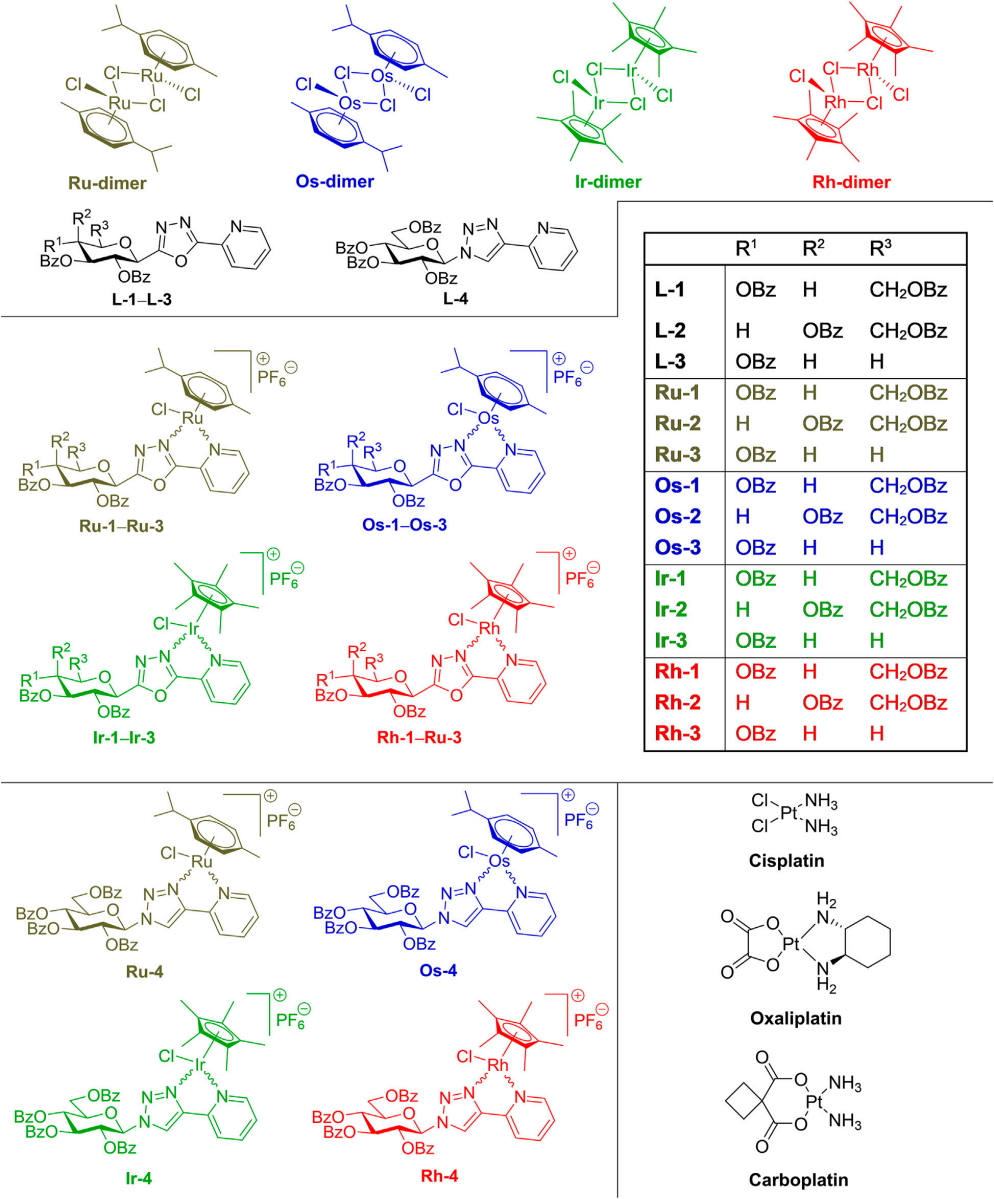
Selected compounds to be tested for antimicrobial activity.

We synthesized a set of half-sandwich complexes of ruthenium(II), osmium(II), iridium(III) and rhodium(III) incorporating real *C*- and *N*-glycopyranosyl azole type N,N-bidentate ligands (Figure 1) (Kacsir et al., 2021; Kacsir et al., 2022). To get the 1,3,4-oxadiazole type **L**-**1**–**L**-**3** ring-transformation of *C*-glycosyl tetrazoles I with picolinic acid was performed (Bokor et al., 2017), while for 1,2,3-triazole-based chelator **L**-**4** copper(I) catalyzed azide alkyne cycloadditon (CuAAc) (Agrahari et al., 2021) of glucosyl azide II was used. The ligands were reacted with dimeric chloro-bridged platinum-group metal complexes in the presence of TlPF_6_ to result in complexes **Ru-1‒Ru-4**, **Os-1‒Os-4**, **Ir-1‒Ir-4** and **Rh-1‒Rh-4** (Figures 1, 2). These complexes were identified to show cytostatic properties on carcinomas (representative data listed in Table 1), sarcomas and lymphomas in the low micromolar or submicromolar range, but have no bioactivity on primary, non-transformed fibroblasts (Kacsir et al., 2021; Kacsir et al., 2022). The compounds exert their cytostatic activity through inducing oxidative stress (Kacsir et al., 2021; Kacsir et al., 2022). The cytostatic activity of the compounds can be alleviated by vitamin E, an apolar, membrane antioxidant (Kacsir et al., 2021; Kacsir et al., 2022) suggesting that the compounds likely target the cell membrane or other apolar compartments in the cells. On the analogy of the bacteriotoxic activity of platinum or palladium compounds (Quirante et al., 2011; Vieites et al., 2011; Zhang et al., 2011; McCarron et al., 2012; Yuan et al., 2018; Hummell and Kirienko, 2020; Yufanyi et al., 2020; Frei et al., 2021; Mansour, 2021) we set out to assess whether the above cytostatic complexes **Ru-1‒Ru-4**, **Os-1‒Os-4**, **Ir-1‒Ir-4** and **Rh-1‒Rh-4** in Figure 2 (Kacsir et al., 2021; Kacsir et al., 2022), might have bacteriostatic properties. For comparative studies, the precursors of these complexes (Kacsir et al., 2021; Kacsir et al., 2022), such as the chloro-bridged platinum-metal dimeric complexes (**Ru-dimer**, **Os-dimer**, **Ir-dimer** and **Rh-dimer**) and the glycosyl heterocyclic N,N-bidentate ligands (**L-1‒L-4**), as well as, the reference platinum-based anticancer drugs (cisplatin, carboplatin, oxaliplatin) were also planned to be tested (Figure 2).

**TABLE 1:** The IC_50_ values [(µM)] of the selected compounds on A2780 ovarian cancer cells in (Kacsir et al., 2021; Kacsir et al., 2022).

**L-1**	**L-2**	**L-3**	**L-4**	**Ru-Dimer**	**Os-Dimer**	**Ir-Dimer**	**Rh-Dimer**
ND	ND	ND	ND	ND	ND	ND	ND
**Ru-1**	**Ru-2**	**Ru-3**	**Ru-4**	**Os-1**	**Os-2**	**Os-3**	**Os-4**
6.2	4.3	8.5	0.9	2.5	3.2	2.8	0.7
**Ir-1**	**Ir-2**	**Ir-3**	**Ir-4**	**Rh-1**	**Rh-2**	**Rh-3**	**Rh-4**
ND	ND	ND	1.6	ND	ND	ND	25.3
Cisplatin		Oxaliplatin		Carboplatin		ND: no effect	
1.2		0.1		28.0			

## Materials and Methods

### Chemical Compounds

All compounds (including cisplatin, carboplatin and oxaliplatin) were from Sigma-Aldrich (St. Louis, MO, United States). Ligands **L-1‒L-4**, complexes **Ru-1‒Ru-4**, **Os-1‒Os-4**, **Ir-1‒Ir-4**, **Rh-1‒Rh-4** were published in (Kacsir et al., 2021; Kacsir et al., 2022). The **Os-dimer** was published in (Godó et al., 2012), **Ru-dimer** was from Strem Chemicals (Newburyport, MA, United States), **Ir-dimer** was from Acros Organics (Gael, Belgium) and the **Rh-dimer** was from Alfa Aesar (Ward Hill, MA, United States). Compounds were dissolved in DMSO. In experiments the highest DMSO concentration was 0.04%, therefore, control cells were treated with 0.04% DMSO.

### Synthesis of the Compounds Tested

Synthesis and assessment of structural integrity of the sugar-based compounds (**L-1‒L-4, Ru-1‒Ru-4, Os-1‒Os-4, Ir-1‒Ir-4, Rh-1‒Rh-4** used in the manuscript (Figures 1, 2) were described in (Kacsir et al., 2021) and (Kacsir et al., 2022).

### Reference Strains

For testing we used the following reference strains: *Pseudomonas aeruginosa* (ATCC27853), *Escherichia coli* (ATCC25922), *Staphylococcus aureus* (ATCC11007), *Candida albicans* (SC5314), *Candida auris* (ATCC21092) and *Enterococcus faecalis* (ATCC29112). All were purchased from ATCC (Manassas, VA, United States).

### Clinical Isolates of *S. aureus* and *E. Faecium*

We used a set of clinical isloates of *S. aureus and E. faecium* that were collected at the Medical Center of the University of Debrecen (Hungary) between 01.01.2018. ‒ 31.12.2020. (Table 2). We also included a multiresistant clinical isolate of *Acinetobacter baumannii.* These were identified using a Microflex MALDI-TOF mass spectrometer (Bruker, Billerica, MA, United States). Antibiotic susceptibility of the isolates was tested following the European Committee on Antimicrobial Susceptibility Testing (EUCAST, 2021) guidelines valid at the time of collection.

**TABLE 2:** Clinical isolates used in the study: MSSA–methicillin-susceptible *Staphylococcus aureus*, MRSA–methicillin-resistant *Staphylococcus aureus*, VSE–vancomycin-susceptible *Enterococcus*, VRE - vancomycin-resistant *Enterococcus*.

	Species		Year	Sample
20276	*S. aureus*	MSSA	2018	Wound
20478	*S. aureus*	MSSA	2018	Bronchial
20559	*S. aureus*	MSSA	2018	Wound
20627	*S. aureus*	MSSA	2018	Ear
20650	*S. aureus*	MSSA	2018	Nasal
20904	*S. aureus*	MSSA	2018	Abscess
20426	*S. aureus*	MRSA	2020	Blood
24035	*S. aureus*	MRSA	2018	Wound
24268	*S. aureus*	MRSA	2018	Throat
24272	*S. aureus*	MRSA	2018	Throat
24328	*S. aureus*	MRSA	2018	Throat
24408	*S. aureus*	MRSA	2018	Bronchial
28046	*E. faecium*	VSE	2021	Abdominal
28386	*E. faecium*	VSE	2021	Urine
25051	*E. faecium*	VRE	2018	Nephrostoma
25342	*E. faecium*	VRE	2021	Urine
25498	*E. faecium*	VRE	2018	Rectal swab for screening for multiresistant pathogens
27085	*E. faecium*	VRE	2018	Wound
28209	*E. faecium*	VRE	2021	Urine
28085	*E. faecium*	VRE	2021	Urine

### Broth Microdilution

Microdilution experiments were performed according to the standards of EUCAST (EUCAST, 2021). The bacterial isolates to be tested were grown in Mueller-Hinton broth. *Candida* species were grown in RPMI (Roswell Park Memorial Institute) -1,640 medium. Inoculum density of bacteria or fungi was set at 5.0 × 10^5^ CFU/ml in microtiter plates in a final volume of 200 µl Mueller-Hinton broth (for bacteria) or in RPMI (for fungi). Tested concentration range was 0.08–40 µM (10 concentrations, two-fold serial dilutions), drug-free growth control and inoculum-free negative control were included. The inoculated plates were incubated for 24 h at 37°C then were assessed visually. Minimum inhibitory concentration (MIC) was defined as the lowest concentration with 50% ≤ inhibitory effect. All experiments were performed at least twice in duplicates.

## Results

### The Complexes Can Inhibit the Growth of Gram-Positive Bacteria

**FIGURE 3:**
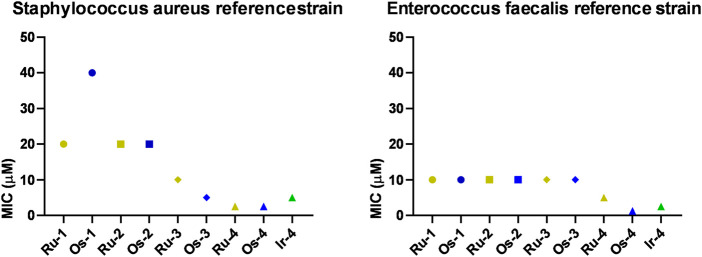
The effects of the complexes on the reference strains of *S. aureus* (ATCC11007) and E. *faecalis* (ATCC29112). Bacterial reference strains were subjected to microdilution assays (repeated at least twice in duplicates) as described in Materials and Methods.

First, we tested the ruthenium(II), osmium(II), iridium(III) and rhodium(III) complexes (**Ru-1‒Ru-4, Os-1‒Os-4, Ir-1‒Ir-4, Rh-1‒Rh-4**; Figure 2) identified in the studies by Kacsir et al. (2021); Kacsir et al., 2022). These compounds were not active on the reference strains of Gram-negative bacteria, such as *Pseudomonas aerugonisa* (ATCC27853), *Escherichia coli* (ATCC25922), or a clinical isolate of *Acinetobacter baumannii*, nor on fungi as *Candida albicans* (SC5314) and *Candida auris* (ATCC21092). Nevertheless, the Gram-positive *Staphylococcus aureus* (ATCC11007) and *Enterococcus faecalis* (ATCC29112) were susceptible to **Ru-1, Os-1, Ru-2, Os-2, Ru-3, Os-3, Ru-4, Os-4** and **Ir-4**, the best being osmium and ruthenium complexes and the complexes of the free ligand **L-4** (Figure 3). Cisplatin, carboplatin and oxaliplatin were included in the study as controls, as they were reported to have antibacterial activity (Zhang et al., 2011; McCarron et al., 2012; Yuan et al., 2018; Hummell and Kirienko, 2020). Cisplatin inhibited the growth of *P. aurigenosa* at a high MIC value of 40 μM, carboplatin and oxaliplatin had no effect suggesting that the effects of platinum complexes were fundamentally different from that of the organometallic bidentate complexes. Neither the free ligands (**L-1‒L-4**), the Ru(II)/Os(II) hexahapto *p*-cymene dimer (**Ru-dimer** and **Os-dimer**), or the Rh(III)/Ir(III) pentahapto arenyl dimer (**Ir-dimer** and **Rh-dimer**), **Ir-1‒Ir-3** and **Rh-1‒Rh-4** complexes had any bacteriostatic activity.

### Complexes Are Active on Multiresistant *Staphylococcus aureus* and *Enterococcus isolates*

**FIGURE 4:**
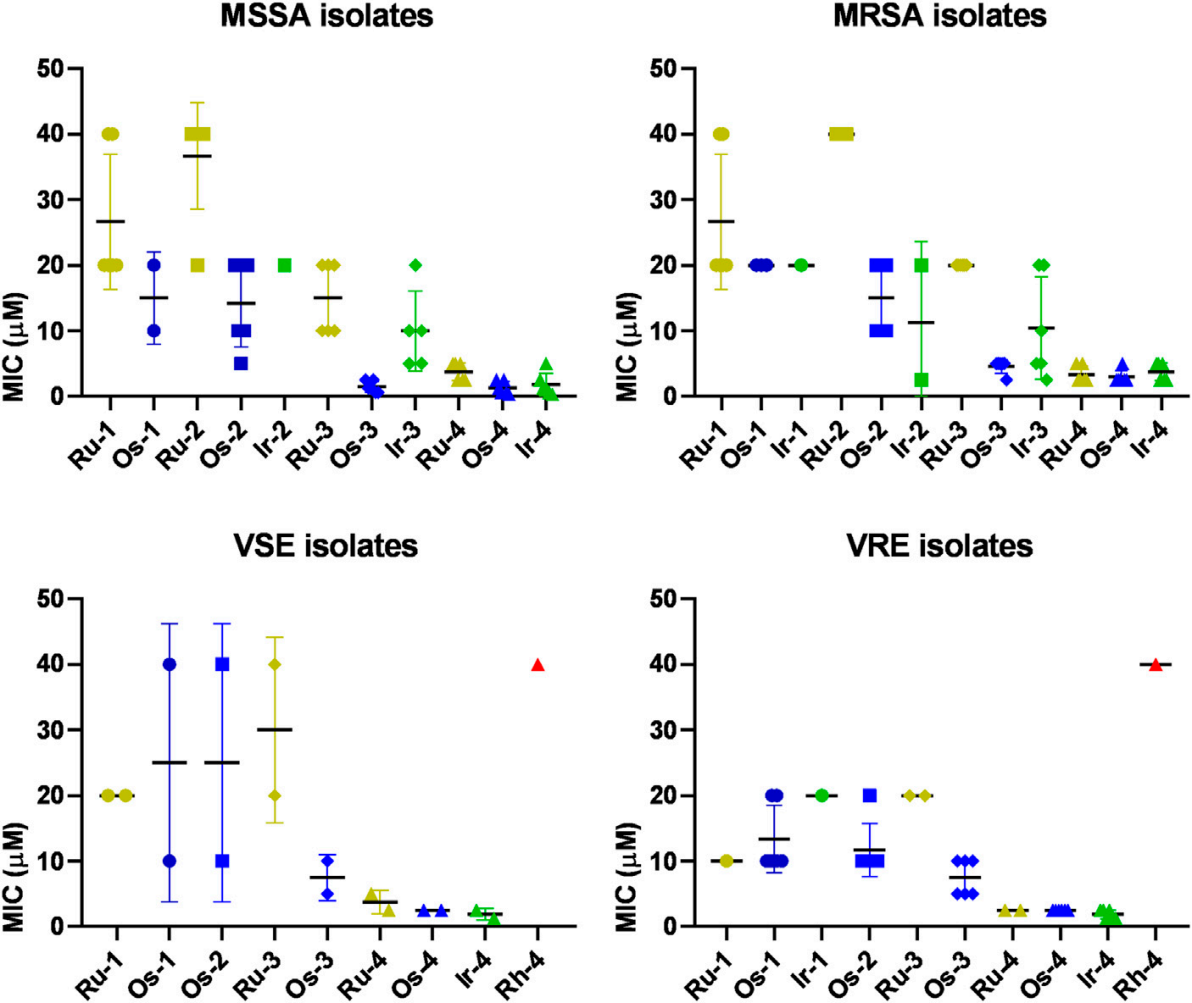
The effects of the complexes on clinical isolates of *S. aureus* and E. *faecium*. MICs were determined by microdilution assays (repeated at least twice in duplicates) as described in Materials and Methods. Abbreviations: MSSA–methicyllin-susceptible *Staphylococcus aureus*, MRSA–methicyllin-resistant *Staphylococcus aureus*, VSE–vancomycin-susceptible *Enterococcus*, VRE–vancomycin-resistant *Enterococcus*.

**FIGURE 5:**
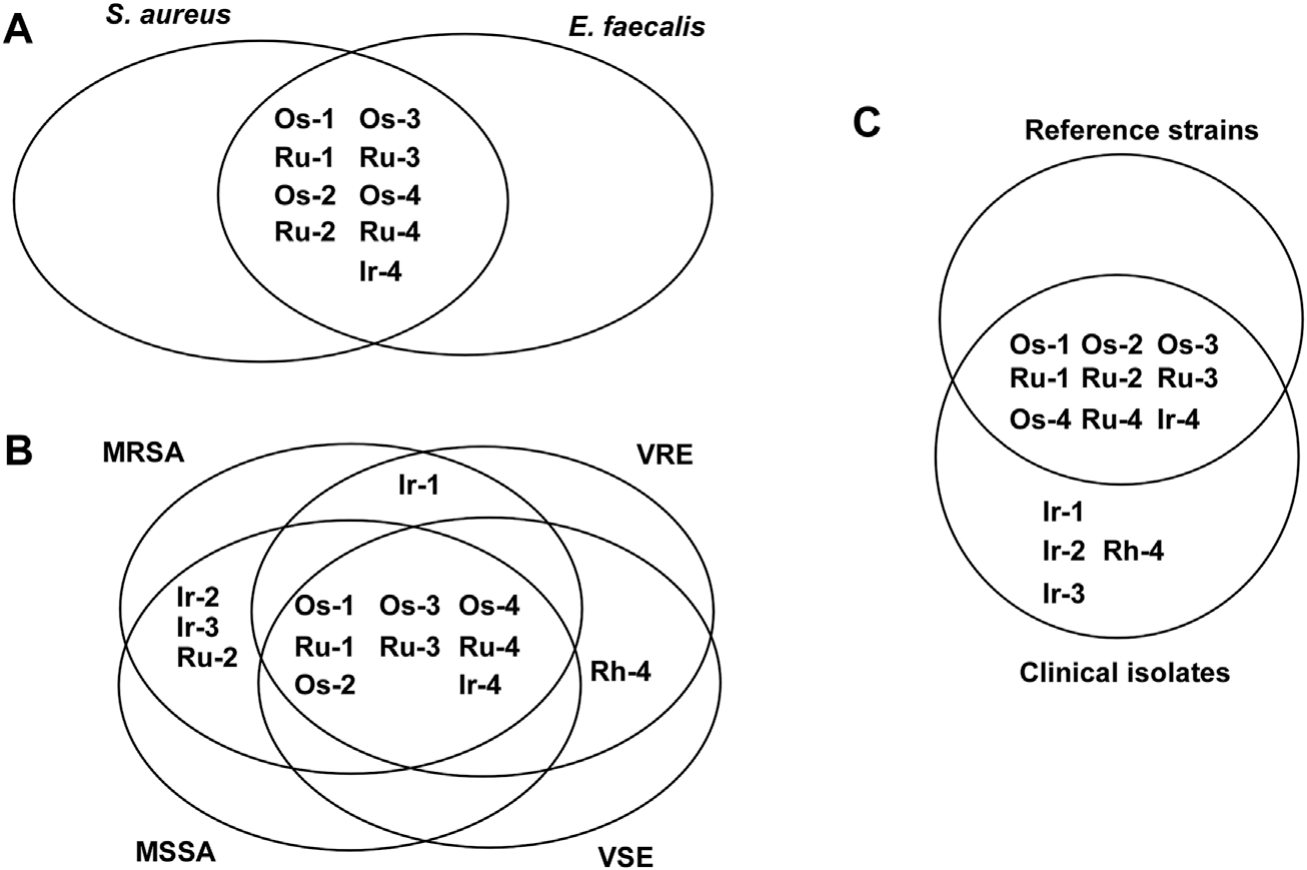
Effects of the complexes on the reference strains and clinical isolates.

Subsequently, we assessed whether the compounds were active on the clinical isolates of *Staphylococcus aureus* [6 methicillin susceptible (MSSA) and six methicillin resistant (MRSA)] and *Enterococcus* sp [2 vancomycin susceptible (VSE) and six vancomycin resistant (VRE)] (Figure 4; Tables 3, 4, 5, 6). MSSA, MRSA, VSE and VRE growth was inhibited by the complexes **Os-2‒Os-4** and **Ir-4** in all isolates (Figures 4, 5; Tables 3, 4, 5, 6). **Ir-1‒Ir-3**, **Ru-1‒Ru-4** and **Os-1** were active only on a subset of isolates (Figures 4, 5; Tables 3, 4, 5, 6). The best activity was observed for the osmium, ruthenium and iridium complexes of **L-4** (**Os-4, Ru-4, Ir-4**) showing MIC values in the low micromolar range (MIC<10 µM) and being active on most or all clinical isolates tested, as well as, on the reference strains (Figures 3, 4; Tables 3, 4, 5, 6). **Rh-4** was active only on *Enterococcus* isolates (both VSE and VRE), but not on MSSA or MRSA isolates (Figures 4, 5; Tables 3, 4, 5, 6). **Rh-1‒Rh-3** complexes were inactive (Figures 4, 5; Tables 3, 4, 5, 6).

**TABLE 3:** MIC values [(µM)] of the complexes against MSSA isolates.

Strain	Ru-1	Os-1	Ru-2	Os-2	Ir-2	Ru-3	Os-3	Ir-3	Ru-4	Os-4	Ir-4
20627	20	>40	40	10	20	20	1.25	5	2.50	0.3	0.60
20559	20	>40	40	20	>40	10	0.60	10	5	0.60	1.25
20650	40	20	40	20	>40	20	0.60	5	5	0.60	0.30
20904	40	>40	40	20	>40	20	2.50	>40	5	2.5	5
20276	20	10	20	5	>40	10	1.25	10	2.50	1.25	1.25
20478	20	>40	40	10	>40	10	2.50	20	2.50	2.50	2.50

**TABLE 4:** MIC values [(µM)] of the complexes against MRSA isolates.

Strain	Ru-1	Os-1	Ir-1	Ru-2	Os-2	Ir-2	Ru-3	Os-3	Ir-3	Ru-4	Os-4	Ir-4
20426	40	20	>40	40	20	>40	20	5	5	2.50	5	5
24408	20	>40	>40	40	10	2.5	20	2.5	2.5	2.50	2.50	2.50
24268	40	20	20	>40	10	>40	20	5	5	2.50	2.50	2.50
20328	20	>40	>40	40	20	>40	20	5	20	2.50	2.50	5
24272	20	20	>40	>40	20	20	20	5	10	5	2.50	2.50
24035	20	>40	>40	40	10	>40	20	5	20	5	2.50	5

**TABLE 5:** MIC values [(µM)] of the complexes against VSE isolates.

Strain	Ru-1	Os-1	Os-2	Ru-3	Os-3	Ru-4	Os-4	Ir-4	Rh-4
28386	20	10	10	20	5	2.50	2.50	1.25	40
28046	20	40	40	40	10	5	2.50	2.50	>40

**TABLE 6:** MIC values [(µM)] of the complexes against VRE isolates.

Strain	Ru-1	Os-1	Ir-1	Os-2	Ru-3	Os-3	Ru-4	Os-4	Ir-4	Rh-4
25051	>40	10	>40	10	>40	10	>40	2.50	1.25	>40
25085	>40	10	>40	10	>40	10	>40	2.50	1.25	>40
25498	>40	10	>40	10	>40	5	>40	2.50	2.50	>40
25342	>40	20	>40	20	>40	5	>40	2.50	2.50	>40
28209	>40	20	20	10	20	5	2.50	2.50	2.50	40
28085	10	10	>40	10	20	10	2.50	2.50	1.25	>40

## Discussion

We assessed a set of half-sandwich type ruthenium(II), osmium(II), rhodium(III) and iridium(III) complexes of monosaccharide derivatives bearing bidentate N,N-chelating sets. The compounds discussed in the study and compounds with similar structure were identified earlier as anticancer agents (Florindo et al., 2014; Florindo et al., 2015; Florindo et al., 2016; Hamala et al., 2020; Kacsir et al., 2021; Kacsir et al., 2022). From the perspective of the current study it is important to note that the complexes were not active on primary human fibroblasts up to 33.3 µM (i.e., their IC_50_ values were higher than 33.3 µM), but only had activity on neoplastic cell lines in low micromolar to submicromolar range (Kacsir et al., 2021; Kacsir et al., 2022) and here we show that these compounds have antimicrobial effects. These suggest that the complexes would be selective towards bacteria and neoplastic cells, which may be an advantageous feature in a clinical setting. The type of the central metal ion and the ligands, the stability and kinetic behavior as well as hydrolytic properties and the lipophilicity of a complex contribute significantly to its biological activity.

With respect to the central metal ion of the complex, osmium compounds were the most efficient on bacteria, followed by ruthenium complexes both in terms of the number of active complexes, as well as, their MIC values, while the iridium and rhodium complexes showed less activity. These findings are similar to our data on cancer cells (Kacsir et al., 2022). In other words, when comparing the Ru(II) and Os(II) complexes with hexahapto *p*-cymene ligand to the pentahapto arenyl-containing Ir(III) and Rh(III) complexes, the former ones were found to show better activity. There are multiple chemical features that can explain this finding. For the mentioned two pairs of metal ions in Ru and Os complexes, the hexahapto coordinated *p*-cym ligand provides less electron densitiy than the Cp* arenyl in the corresponding Rh or Ir compounds and their steric hindrance is different. Kinetic differences may also provide an explanation, as it is widely accepted that the half-sandwich type Os and Ir complexes, in general, exhibit much lower ligand exchange rates than the Ru and Rh analogues (Bruijnincx and Sadler, 2009). When comparing the IC_50_ values for cancer cells with the MIC values against bacteria, it is apparent that the MIC values of the active complexes are higher than their IC_50_ values on the most sensitive cancer cell model [e.g., for **Os-4** IC_50_ = 0.7 µM on 2780 ovarian cancer cells (Kacsir et al., 2021; Kacsir et al., 2022) vs. MIC range = 0.3–5 µM on multiresistant bacteria]. When comparing the MIC values of the complexes we found similar trends as a function of the central metal ion or the ligand as the IC_50_ values of the complexes on cancer cells. Complexes of **L-4** were considerably more effective than complexes of **L-3, L-2** or **L-1**. These findings are also in good correlation with our observations on cancer cells (Kacsir et al., 2021; Kacsir et al., 2022) and may support the importance of the high hydrophobicity of the complexes. Importantly, for the complexes with good bacteriostatic activity (e.g., **Os-4**) there was no difference in the MIC value on the reference strains, the susceptible (MSSA, VSE) or the multiresistant isolates (MRSA, VRE). The activity of the complexes in previous antineoplastic studies was dependent on the apolar character of the compounds (Kacsir et al., 2021; Kacsir et al., 2022). We provided experimental evidence the carbohydrate moiety has a key role in bringing about the apolar character of the molecules by harboring multiple OBz groups. The replacement of the carbohydrate moiety with one single aromatic group largely hampered or eliminated the biological activity of the complexes (Kacsir et al., 2021). Therefore, the complexes supposedly affect the cell membrane that may be the case in bacteria as well. It is also of note that the exact target of the complexes has not been identified yet. Taken together, we identified osmium, ruthenium, iridium and rhodium complexes that exhibit antibacterial effects. The complexes have multiple advantageous properties, they are stable over extended periods [2 days were assessed in (Kacsir et al., 2021)], their MIC and IC_50_ values are in the low micromolar or submicromolar range, respectively, and they are not active on non-transformed cells. As noted earlier, the active complexes have similar MIC values against multiresistant clinical isolates of MRSA and VRE and on sensitive isolates or reference strains suggesting a novel, yet unidentified target in Gram-positive bacteria that is not detoxified by existing resistance mechanisms. These findings suggest that the complexes studied here and similar ones may represent a novel class of antibiotics against multiresistant Gram-positive bacteria.

## Data Availability

The original contributions presented in the study are included in the article, further inquiries can be directed to the corresponding authors.
